# Vertical Structure of Phyllosphere Fungal Communities in a Tropical Forest in Thailand Uncovered by High-Throughput Sequencing

**DOI:** 10.1371/journal.pone.0166669

**Published:** 2016-11-18

**Authors:** Ayako Izuno, Mamoru Kanzaki, Taksin Artchawakom, Chongrak Wachrinrat, Yuji Isagi

**Affiliations:** 1 Graduate School of Agriculture, Kyoto University, Kyoto, Japan; 2 Sakaerat Environmental Research Station, Nakhon Ratchasima, Thailand; 3 Faculty of Forestry, Kasetsart University, Bangkok, Thailand; Leibniz Institut—Deutsche Sammlung von Mikroorganismen und Zellkulturen GmbH, GERMANY

## Abstract

Phyllosphere fungi harbor a tremendous species diversity and play important ecological roles. However, little is known about their distribution patterns within forest ecosystems. We examined how species diversity and community composition of phyllosphere fungi change along a vertical structure in a tropical forest in Thailand. Fungal communities in 144 leaf samples from 19 vertical layers (1.28–34.4 m above ground) of 73 plant individuals (27 species) were investigated by metabarcoding analysis using Ion Torrent sequencing. In total, 1,524 fungal operational taxonomic units (OTUs) were detected among 890,710 reads obtained from the 144 leaf samples. Taxonomically diverse fungi belonging to as many as 24 orders of Ascomycota and 21 orders of Basidiomycota were detected, most of which inhabited limited parts of the lowest layers closest to the forest floor. Species diversity of phyllosphere fungi was the highest in the lowest layers closest to the forest floor, decreased with increasing height, and lowest in the canopy; 742 and 55 fungal OTUs were detected at the lowest and highest layer, respectively. On the layers close to the forest floor, phyllosphere fungal communities were mainly composed of low frequency OTUs and largely differentiated among plant individuals. Conversely, in the canopy, fungal communities consisted of similar OTUs across plant individuals, and as many as 86.1%–92.7% of the OTUs found in the canopy (≥22 m above ground) were also distributed in the lower layers. Overall, our study showed the variability of phyllosphere fungal communities along the vertical gradient of plant vegetation and environmental conditions, suggesting the significance of biotic and abiotic variation for the species diversity of phyllosphere fungi.

## Introduction

Phyllosphere fungi, which inhabit tissues of living plants, have been recognized to harbor tremendous species diversity and play significant ecological roles [1]. Almost all major lineages of land plants distributed from polar regions to tropics are associated with taxonomically diverse phyllosphere fungi [1–3] and can be subject to some ecological effects by these fungi, such as pathogenic damage [4, 5] or benefits of enhancing tolerances against herbivores or pathogens [6–8]. However, many phyllosphere fungi, including epiphytes and endophytes, have cryptic lifestyles with transient settling and neutral influences on the host plants. Thus, the ecological functions as well as species diversity and community structure of phyllosphere fungi have been difficult to determine [1]. Recently, with metabarcoding analysis using high-throughput sequencers, species diversity and community structure of phyllosphere fungi, including rare and cryptic species, has been successfully revealed in various terrestrial ecosystems [9–11]. Accumulative studies on species diversity and community structure of phyllosphere fungi in forests–which present the ecosystem with the greatest biomass, productivity, and species diversity on Earth–can contribute to the detection of novel bio-resources, clarification of ecological interactions between fungi and host plants, and control of pathogenic fungi. All these aspects are associated with the management and conservation of forest ecosystems.

In tropical forests, the forest canopy is characterized by biotic and abiotic conditions that are largely different from those in the understory. The forest canopy bears the majority of the productivity [12] and influences the hydrology of forests through intercepting precipitation and controlling evapotranspiration [13]. The forest canopy is referred to as “the last biological frontier,” as it harbors a diverse but poorly studied assemblage of plant and animal species [14, 15], including canopy-specific species [16]. The forest canopy houses a significant proportion of the flora and fauna in a forest, and further knowledge of the forest canopy ecosystem including the fungal flora associating with plant or animal species is needed to determine strategies for forest conservation and management [17, 18]. We have little knowledge about whether the fungal flora in the canopy is distinctive from that in the understory and the proportion of fungal species richness the canopy harbors within a forest.

In a mature forest, multiple coexisting plant individuals of various species and ages compete with each other to occupy specific niches. Many of the niches in the canopy within a forest are created by the spatial arrangement of plant leaves [19]. The vertical stratification of plant leaves forms structural complexities, resulting in temporal and spatial variability of resources and microclimatic conditions within a forest [20, 21]. Environmental conditions have been recognized to significantly affect the assemblage of phyllosphere fungi. Community compositions of phyllosphere fungi have been shown to be significantly different among environmental variations along elevation [9, 11, 22, 23], landscape [10], climatic condition of a continent [24] and across latitudes [25, 26], and season [27–29]. Therefore, we predict that species richness and community compositions of phyllosphere fungi differentiate among heights within a forest. The distribution patterns of phyllosphere fungi on a vertical scale within a forest from ground to the canopy are expected to provide insights into habitat preference or spatial dynamics of phyllosphere fungi associated with plants.

In the current study, we investigated the vertical structure of phyllosphere fungal communities in a tropical forest in Thailand. Fungal community compositions in leaf samples, collected from 144 spatial locations distributed within a 314 m^2^ area × 34.4 m height, were assessed with metabarcoding using the Ion Torrent sequencer. We studied the vertical changes of (1) the occurrence of fungal taxa, (2) species diversity, and (3) community compositions of phyllosphere fungi within a forest.

## Materials and Methods

### Study site and plant materials

**Fig 1:**
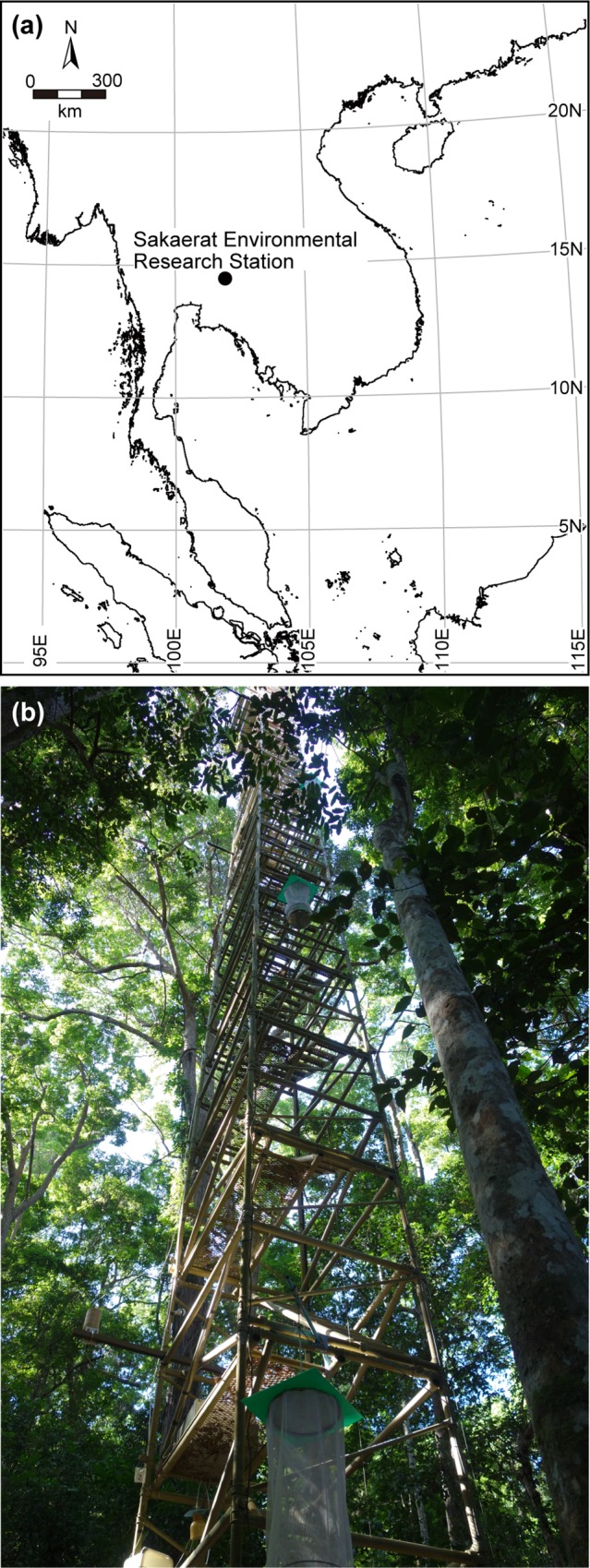
Map of the study site and the tower used for leaf sampling. (a) Location of Sakaerat Environmental Research Station, Thailand. (b) The 40-m steel tower used for leaf sampling.

**Fig 2:**
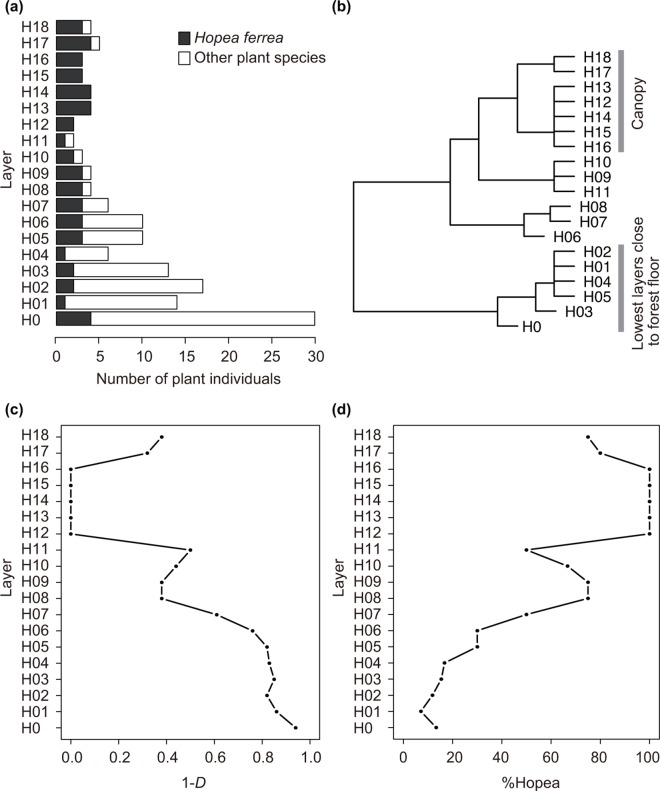
Summary of the vertical stratification of host plants. (a) The total number of samples in each vertical layer is shown in white. *Hopea ferrea*, a dominant tree species at the site, is shown in black. (b) Ward clustering of the Chao dissimilarity of vegetation among the 19 vertical layers. The canopy layers and the lowest layers close to the forest floor were represented by H0–H05 and H12–H18, respectively. (c) Simpson’s diversity index (1-*D*) of host plants in each vertical layer. (d) Percentage of *Hopea ferrea* samples (%Hopea) in each vertical layer.

Leaves were collected in November 2012 in a tropical seasonal evergreen forest at Sakaerat Environmental Research Station in Nakhon Ratchasima Province, northeast Thailand, approximately 180 km northeast of Bangkok (14°30´N, 101°51´E; Fig 1A). The climate of the site is monsoonal: mean annual temperature and precipitation of the site are 26.2°C and 1,240 mm, respectively, and the dry season occurs from December to February with the monthly mean precipitation lower than 50 mm [30–32]. The seasonal evergreen forest, covering 53% of the total area of the research station [32], is characterized by the dominant tree species *Hopea ferrea* Laness (Dipterocarpaceae). Using a 40-m steel tower with ladders (Fig 1B) located on a flat site with an elevation of 600 m above sea level and a telescopic pruning pole, leaves were collected from 73 individuals of 27 plant species growing within a 10-m radius from the tower, at 19 layers on the vertical dimension (labeled H0–H18 from the ground level to the top) (Fig 2A; S1 Fig). Leaves were collected from all 43 plant individuals found within the 10-m radius at H01–H18, ranging from 1.28 m to 34.4 m above the ground, with intervals of 1.84 m (distance between consecutive floors of the tower) (Fig 2A; S1 Fig). The locations of the 43 plant individuals were recorded using a LaserAce^TM^ 1000 rangefinder (Trimble, Sunnyvale, CA, USA). Because the 43 studied plants bore no leaves at level H0, we randomly selected 30 plants as targets for this level, and collected leaves at the height of 1 m from the ground. At each vertical layer, three leaves without any evident symptoms of a pathogenic fungal infection were collected from one plant individual. In total, 144 sampling points × three leaves were collected (Fig 2A; S1 Fig) and dried using silica gel until DNA extraction. The study was conducted under the specific agreement between the Thailand Institute of Scientific and Technological Research and the Graduate School of Agriculture, Kyoto University. The research permission was issued under the responsibility of the National Research Council of Thailand.

### Vertical stratification of host plants

In each vertical layer, the species diversity of plant individuals from which leaves were collected was evaluated with Simpson’s diversity index (1-*D*) and the percentage of *H*. *ferrea* samples among the entire plant individuals (%Hopea). The similarity of vegetation among vertical layers was investigated with the Chao dissimilarity index and Ward clustering.

### DNA extraction for fungal metabarcoding

A 25-mm^2^ portion was cut from each leaf sample using a disposable knife, and the three leaf pieces from each sampling point were mixed in a microcentrifuge tube. From this point in the current document, a single sample is defined as the mix of the three leaf pieces originating from a single plant individual collected at a sampling point. To avoid cross contaminations between samples during sample collection in the field, transportation, and laboratory work, the leaf pieces were cleaned three times using 0.005% (*w*/*v*) di-(2-ethylhexyl) sodium sulfosuccinate (Aerosol OT) solution and rinsed two times using sterile distilled water [33]. The leaf pieces were then homogenized in a microcentrifuge tube with stainless beads (diameter = 3 mm) until completely powderized, followed by the extraction of total genomic DNA using a modified cetyltrimethylammonium bromide (CTAB) method [34]. Two tubes containing no leaf pieces were prepared as blank samples to detect contaminations during the DNA extraction procedure.

### Parallel amplicon sequencing

The internal transcribed spacer 1 (ITS1) region was amplified by polymerase chain reaction (PCR). To conduct parallel amplicon sequencing using Ion PGM^TM^ (Ion Torrent, Life Technologies, Guilford, CT, USA), we used two types of fusion primers: 1) a PGM-sequencing primer (Ion A primer) followed by tag sequences for sample identification [35] and ITS1F_KYO2 [36] and 2) a DNA capture bead anneal primer for emulsion PCR (trP1 primer) followed by ITS2_KYO2 [36]. Fungal ITS1 regions were amplified in a total volume of 50 μL containing 10 ng of the template DNA, 200 nM of each primer, and 45 μL of Platinum PCR SuperMix High Fidelity (Invitrogen, Life technologies Inc., Burlington, Ontario, Canada). Using a Veriti 96-well Thermal Cycler (Applied Biosystems, Life Technologies, Foster City, CA, USA), amplification reactions were performed under the following conditions: 94°C for 3 min (initial denaturation), 35 cycles at 94°C for 30 s, 60°C for 30 s, and 68°C for 2 min and 40 s.

Amplicons with a length of 300–450 bp were extracted by agarose gel electrophoresis using E-Gel SizeSelect Agarose Gels (Invitrogen). The extracted amplicons were then purified using Agencourt AMPure XP PCR Purification Reagent (AgenCourt Bioscience, Beverly, MA, USA) and quantified using the Agilent 2100 Bioanalyzer^TM^ DNA High Sensitivity Kit (Agilent Technologies, Santa Clara, CA, USA). Amplicons of all samples were mixed and diluted to a final concentration of 20 pM. The amplicon library was amplified using the Ion One Touch^TM^ 2 System (Ion Torrent) with Ion PGM™ Template OT2 400 Kit (Ion Torrent) and then sequenced using an Ion PGM^TM^ (Ion Torrent) with an Ion PGM™ Sequencing 400 Kit (Ion Torrent) and an Ion 318^TM^ Chip v2 (Ion Torrent). Out of the 144 samples, 113 samples plus a blank sample were sequenced in the first run, and the remaining 31 samples plus another blank sample were sequenced in the second run (S1 Table). In the second run, we included an extra 113 leaf samples, which were collected in the different sites in the research station and not related to the present study. In total, 6,126,561 and 5,134,841 reads were obtained from the first and second run, respectively.

### Sequence processing and taxonomic identification

After each Ion-PGM sequencing run, the raw sequence reads were sorted according to the sample-specific tag sequences. In total, 4,084,221 and 891,211 reads were obtained from the 114 and 32 samples, respectively (DDBJ Sequence Read Archive (DRA) accession: DRA002469). The following sequence processing was performed with these reads. Reads with less than 150 bp or average Phred quality score below 30 were excluded using the “clfilterseq” command in the Claident v0.2.2015.11.19 software package [37]. Then, potentially chimeric or erroneous reads were detected and removed by running UCHIME v4.2 [38] called by the “clcleanseq” command in Claident. After these filtering and denoising processes, 785,580 and 196,785 reads were retained for the 114 and 32 samples, respectively. The number of retained reads per sample was not different between the first and second sequence run (*p* = 0.14, Welch’s *t*-test). The average length of the retained sequences was 233 bp and 214 bp in the first and second sequence run, respectively. Although a large number of sequence reads obtained from the high throughput sequencer resulted in a statistically significant difference in length between the two sequence runs (Wilcoxon rank sum test: *p* < 0.001), the raw sequence length was almost the same between the two runs (1–602 bp in the first run and 1–603 bp in the second run) and size-selections were likely conducted equivalently in the two runs.

The filtered and denoised reads were clustered using the Assams v0.2.2015.08.08 software [39, 40], which was operated using the “clclassseq”, “clclassclass” and “clreclassclass” commands in Claident [37]. Assams enables highly parallelized processing using the accurate assembling program Minimus [41]. The reads within each sample were clustered with a cutoff sequence similarity of 99%. The clustered sequences were then merged across all samples with a cutoff sequence similarity of 97% [9, 28, 40]. The resulting sequence clusters were used as operational taxonomic units (OTUs) in the analysis described below. OTU clustering was also performed based on the UPARSE algorithm implemented in USEARCH [42] and qualitatively the same taxonomic compositions were detected.

Taxonomic affiliations of the obtained OTUs were inferred based on the query-centric auto-*k*-nearest-neighbor (QCauto) method [43] operated using the “clidentseq” and “classigntax” commands in Claident v0.2.2015.11.19 [37]. The QCauto method has been shown to return the most accurate taxonomic identification results among existing automated DNA barcoding methods [43]. We queried against the “fungi_ITS_species” database downloaded from web site of the National Center for Biotechnology Information on 15 November 2015, which is the subset of the “nt” database of the NCBI and comprised reference ITS sequences of fungi with species-rank taxonomic information [43]. Claident returns the lowest taxonomic level common to the homologous sequences found in the reference database using the lowest common ancestor algorithm [44]. Sequences of fungal OTUs whose taxonomic affiliations were not identified with Claident were searched among the “Top 50 most wanted” database (ver. 7.1; 53,693 sequences) [45] provided in UNITE (https://unite.ut.ee/sh_files/top50_release_22.08.2016.zip) using BLASTN (ver. 2.2.31+; E-value = 1.0e−15). Taxonomic affiliations were supplementary inferred using the UNITE dynamic database (ver. 7) [46] with the BLAST method implemented in QIIME (E-value = 1.0e−5) [47]. After the taxonomic identification process, singletons, non-fungal OTUs, and those included in the blank samples were excluded from the dataset. A command script for the sequence processing using Claident is provided in S1 Data.

### Data analysis

The spatial structure of phyllosphere fungal communities in the forest was investigated using the two datasets: 1) a total of 144 samples collected from all 27 plant species (73 plant individuals) to analyze the three-dimensional spatial structure of fungal communities in the forest and 2) a total of 51 samples collected from a single dominant plant species *H*. *ferrea* (13 individuals) to analyze the effect of height on the community compositions of phyllosphere fungi inhabiting a single plant species. *H*. *ferrea* appeared in every layer; therefore, in the analysis of the dataset of *H*. *ferrea*, we could evaluate the effect of height on the phyllosphere fungal communities independent of the bias due to the differences in the host plant species. In both datasets, the fungal OTU composition of each plant sample was represented by the presence/absence of each fungal OTU.

#### Vertical changes in taxonomic compositions of phyllosphere fungi

**Fig 3:**
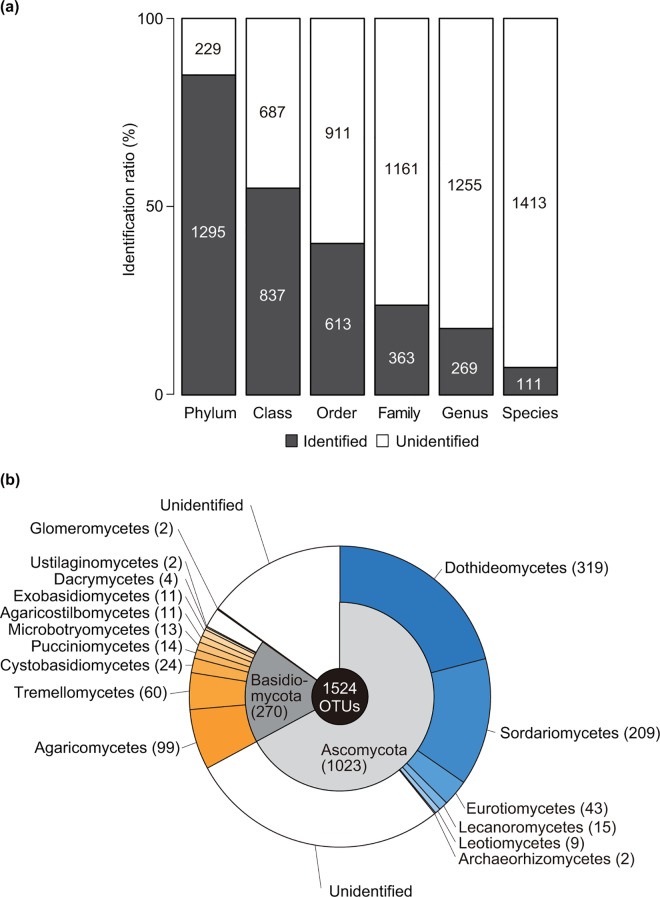
Taxonomic identification of the 1,524 fungal operational taxonomic units (OTUs). (a) Proportion of identified fungal OTUs at each taxonomic rank. (b) Phylum and class level taxonomic composition of the 1,524 fungal OTUs.

OTU abundance in each vertical layer was represented by the total occurrence across all samples found in the layer. The OTU abundances were added for each fungal order or family, and a matrix depicting the OTU abundance in each fungal order or family at each layer (hereafter, “order- or family-level abundance” matrix) was constructed. To examine the differences in spatial distributions among fungal orders, the order-level abundance matrix was illustrated with a two-dimensional heatmap. To identify fungal families specific to the canopy or lowest layers close to the forest floor, the multinomial model based classification method (CLAM) [48] implemented in the R vegan 2.3–3 package [49] was carried out for the family level abundance matrix. Each fungal family was classified into four groups: (1) family preferentially inhabiting the canopy layers (H12–H18; Fig 2B), (2) family preferentially inhabiting the lowest layers (H0–H05; Fig 2B), (3) family commonly inhabiting the canopy and lowest layers, and (4) rare family that cannot be classified into these three groups with confidence.

#### Vertical changes in species diversity of phyllosphere fungi

Species diversity of phyllosphere fungi in each vertical layer was evaluated by two parameters: 1) OTU richness and 2) the evenness of the abundance across fungal OTUs. OTU richness was estimated with observed, rarefied and extrapolated number of OTUs. To correct the difference in sample size among layers, the rarefied number of OTUs was compared at the sample size of 2, 3, and 4. The extrapolated number of OTUs was estimated with Chao [50], first and second-order jackknife richness estimators [51]. The evenness of the OTU occurrence in each layer was evaluated using the rank-abundance curve and the proportion of infrequent OTUs [52]. A frequency of occurrence, which was defined as a proportion of inhabiting samples among all the samples, was plotted against a frequency rank for each layer. OTUs with a frequency of occurrence of less than 0.5 were defined as infrequent OTUs and the remaining OTUs were defined as common OTUs.

#### Vertical structure of phyllosphere fungal communities

The spatial variability of phyllosphere fungal communities in the forest was investigated by the dissimilarity of fungal OTU compositions among all the 144 samples. The difference in fungal OTU compositions among samples was represented by Raup–Crick dissimilarity and Jaccard similarity index. The Raup–Crick dissimilarities were calculated using the “raupcrick” function in the R vegan 2.3–3 package [49]. This function can correct differences in the observed numbers of OTUs among groups and differences in sampling intensities among OTUs [49, 53]. Based on the Raup–Crick dissimilarity or Jaccard similarity index, the fungal community of each sample was ordinated in a three-dimensional space by non-metric multi-dimensional scaling (NMDS) analysis using the vegan 2.3–3 package [49]. Using the “ordisurf” function in vegan [49], the height of the samples was fitted on the NMDS plots. For the NMDS analysis based on the Raup–Crick dissimilarity, the coordinate of each sample on the NMDS1, NMDS2, and NMDS3 axis was then transformed into a hexadecimal value. Combining the hexadecimal values, each fungal community was depicted with red–green–blue (RGB) color values corresponding to the coordinate on the NMDS1, NMDS2, and NMDS3 axis, respectively. Here we aimed to represent the similarity of fungal OTU compositions using colors. By plotting each fungal community with RGB color in a three-dimensional space reflecting the actual spatial location in the forest, the spatial variability of phyllosphere fungal communities was visualized. The homogeneity of fungal communities across samples within layers was evaluated with the multivariate dispersion calculated by the “betaspider” function in the vegan 2.3–3 package [49]. Dispersion of fungal communities within a given layer was represented by the average distance between each fungal communities and the centroid within a layer on the NMDS plot.

To reveal the difference in spatial distribution among fungal OTUs, which could form the spatial variability of phyllosphere fungal communities within the forest, the number of layers in which a fungal OTU was observed was surveyed. A fungal OTU found only at a single layer was then defined as a “layer-specific OTU.” To evaluate the specificity of fungal communities in each vertical layer, the proportion of layer-specific OTUs out of the total OTUs was calculated for each layer. A command script and data used for statistical analyses using R is provided in S3 Data.

## Results

### Vertical stratification of host plants

In the Ward clustering of vegetation, the vegetation in the lowest layers (H0–H05) was different from that in the remaining layers (Fig 2B). The higher layers (H06–H18) were clustered into three groups: H06–H08, H09–H11, and H12–H18 (Fig 2B). Therefore, the canopy layers and the lowest layers were represented by H0–H05 and H12–H18, respectively. The species diversity of plant samples decreased from understory to canopy as shown in the decreasing Simpson’s diversity index (1-*D*) and the increasing percentage of *H*. *ferrea* samples (%Hopea) (Fig 2C and 2D). The vegetation above H08 (≥14.17 m above ground) was mostly dominated by *H*. *ferrea* and showed lower species diversity (Fig 2C and 2D).

### Overall OTU richness and taxonomic compositions of phyllosphere fungi

After clustering the quality-filtered and denoised sequence reads with a 97% similarity cutoff, 1,772 OTUs (982,365 reads) were obtained. Out of the 1,772 OTUs, 241 non-fungal OTUs, and 7 OTUs found in the blank samples were excluded from the dataset. As a result, 1,524 fungal OTUs (890,710 reads) were analyzed (S1 Table; S2 Data). Out of the 7 OTUs found in the blank samples, 4 belonged to Ascomycota and 3 were not identified even at the phylum level. The average number of detected OTUs per sample was 49 ± 35. Out of 1,524 OTUs, 111 OTUs (7.3%) were identified at species level (Fig 3A). In total, phyllosphere fungi within 86 families, 48 orders, 16 classes and three phyla were detected (Fig 3B). In the BLAST against the UNITE dynamic database, taxonomic affiliations for 464 OTUs out of 1,413 unidentified OTUs (Fig 3) were additionally inferred (S2 Table). Hereafter, we mainly discuss the taxonomic compositions in the fungal community in the forest based on the results obtained with the QCauto method, which has been shown to identify fungal taxonomies more correctly and conservatively than the other known methods [43].

**Fig 4:**
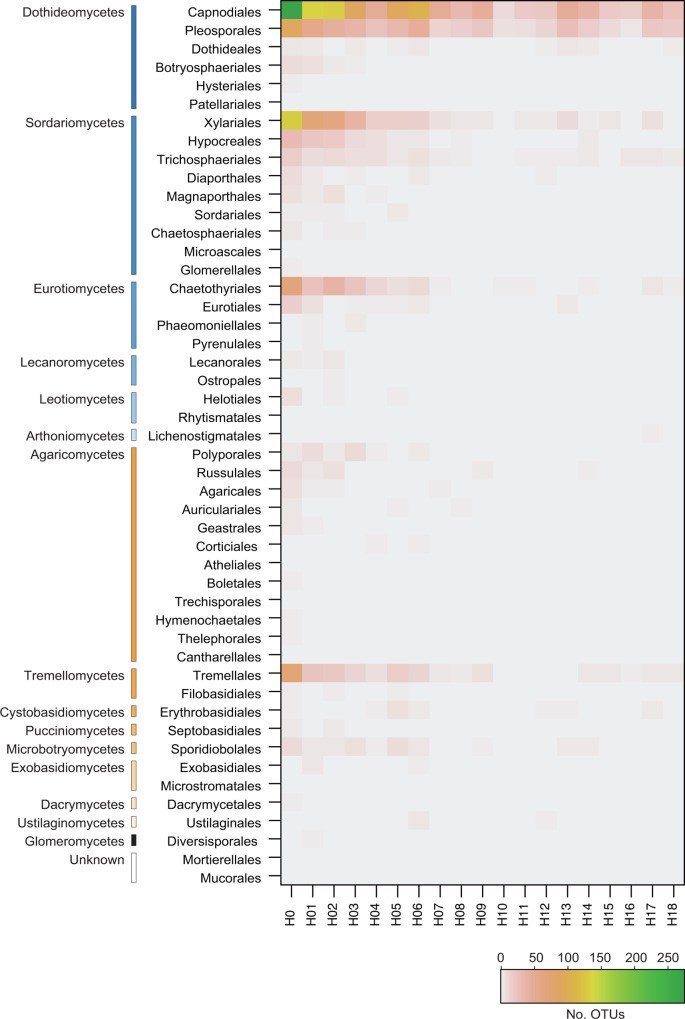
Vertical changes in fungal operational taxonomic unit (OTU) abundance of 48 orders. Out of the 1,524 OTUs obtained from all 144 samples, the occurrence of 613 OTUs which were identified at the order level are shown.

**Fig 5:**
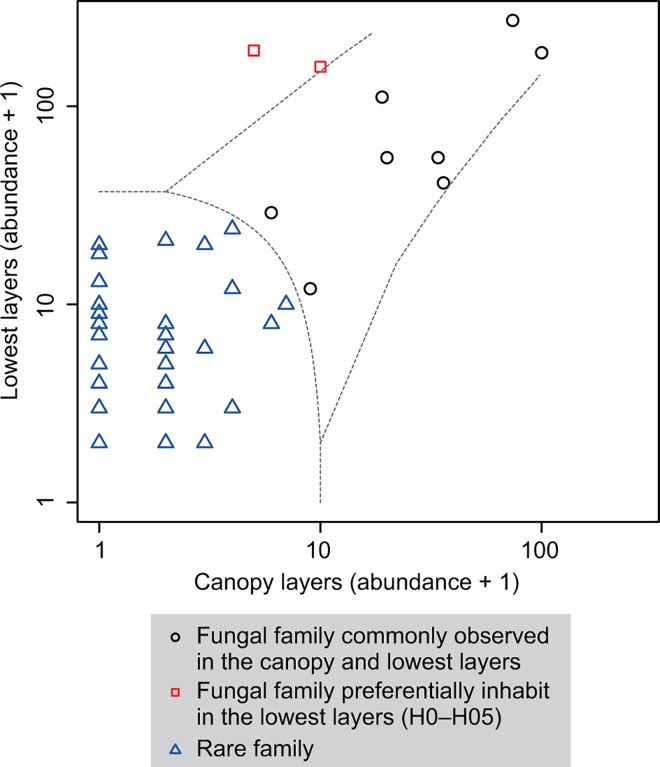
Habitat preference of fungi at the family-level between the canopy (H12–H18) and lowest (H0–H05) layers. The CLAM test classified fungal families into four groups: (1) families preferentially inhabiting the canopy layers (H12–H18; Fig 2B), (2) families preferentially inhabiting the lowest layers (H0–H05; Fig 2B), (3) families commonly inhabiting the canopy and lowest layers, and (4) rare families. Two families (Chaetothyriaceae and Xylariaceae) significantly preferred the lowest layers close to the forest floor, while no family preferentially inhabited the canopy.

Diverse fungal taxa were identified in the 1,524 OTUs. At the phylum level, 1,023 OTUs (67.1%), 279 OTUs (17.7%), and 2 OTUs (0.1%) were identified as Ascomycota, Basidiomycota, and Glomeromycota respectively, and the taxonomic affiliation of the remaining was not confirmed (Fig 3B). At the class level, Dothideomycetes (319 OTUs; 20.9%) dominated, followed by Sordariomycetes (209 OTUs; 13.7%), Agaricomycetes (99 OTUs; 6.5%), and Tremellomycetes (60 OTUs; 3.9%) (Fig 3B). Order compositions of phyllosphere fungal communities changed along the height of trees (Fig 4). In every layer, Capnodiales (Dothideomycetes) dominated, followed by Pleosporales (Dothideomycetes) (Fig 4). However, other fungal orders, particularly Xylariales (Sordariomycetes), Hypocreales (Sordariomycetes), and Tremellales (Tremellomycetes) inhabited mainly the lower layers (H0–H05), and rarely the higher layers (H12–H18) (Fig 4). In the CLAM test, two families (Chaetothyriaceae and Xylariaceae) preferentially inhabited the lowest layers (H0–H05), while no family preferentially inhabited the canopy layers (H12–H18) (Fig 5). The 8 families commonly inhabiting the lowest and canopy layers were Cladosporiaceae, Mycosphaerellaceae (Capnodiales), Phaeosphaeriaceae, Pleosporaceae (Pleosporales), Amphisphaeriaceae (Xylariales), Trichosphaeriaceae (Trichosphaeriales), Aspergillaceae (Eurotiales), and Erythrobasidiaceae (Erythrobasidiales). Among the 229 fungal OTUs whose taxonomic affiliations were not identified at the phylum level with Claident, 216 fungal OTUs were likely listed in the “Top 50 most wanted” database in UNITE (S3 Table).

### Vertical changes in species diversity of phyllosphere fungi

**Fig 6:**
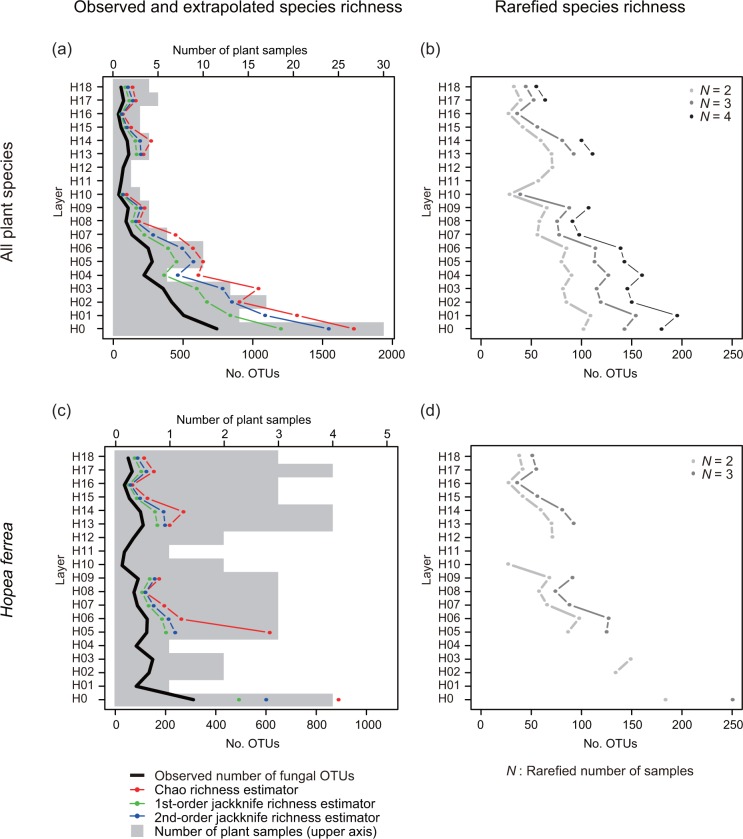
Species richness of phyllosphere fungal operational taxonomic units (OTUs) in each vertical layer. (a), (c) The observed and extrapolated number of OTUs was shown for each vertical layer. Complete OTU richness was estimated with three indices: Chao index (red), first- (green) and second-order jackknife index (blue). Gray bar plots indicate the number of host plant individuals. (b), (d) The rarefied number of OTUs at the sample size of 2, 3, and 4. Two datasets were analyzed: (a), (b) a total of 144 samples collected from all 27 plant species and (c), (d) a total of 51 samples collected from a single tree species, *Hopea ferrea*.

The observed and extrapolated OTU richness decreased along with the height (Fig 6). In H16, H17, and H18, the observed number of OTUs was 36, 74, and 55, respectively, which were approximately 5–10% of that found in H0 (Fig 6A). This pattern was also shown in the rarefied number of OTUs (Fig 6B) and the dataset of *H*. *ferrea* (Fig 6C and 6D). Extrapolated number of OTUs indicated that every layer potentially harbored more species richness than observed: the average proportion of the observed number of OTUs out of the expected number of fungal OTUs with the Chao richness estimator was 42.6% (Fig 6A and 6B).

**Fig 7:**
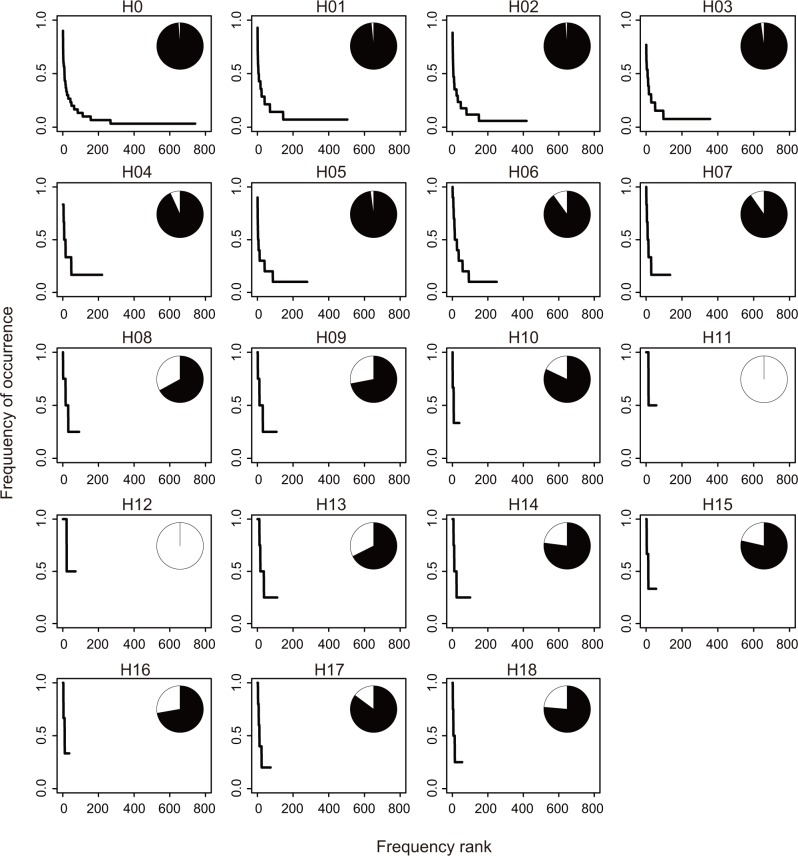
Rank–abundance plot for fungal OTUs found in each vertical layer. For each OTU, the proportion of inhabiting samples out of all samples in the layer (frequency of occurrence) is shown against the rank of the number of inhabiting samples (frequency rank). A total of 144 samples collected from all host plant species were analyzed (see also S2 Fig). Inset pie charts indicate the relative proportion between infrequent (black) and common (white) OTUs. OTUs with a frequency of occurrence of less than 0.5 were defined as infrequent OTUs, and the remaining OTUs were recognized as common OTUs.

The largest variation in the frequency of occurrence of fungal OTUs was found in the lower layers (H0–H05); the average frequency of occurrence was 0.07–0.22 and the proportion of infrequent OTUs was 93.2–98.8% (Fig 7). In the layers above H08, where the number of host plant samples was less than 5, the evenness in the frequency of fungal OTUs was high; the average frequency of occurrence was over 0.32 and the proportion of infrequent OTUs was below 85% (Fig 7). The increased evenness in the frequency of occurrence of fungal OTUs in the higher layers was additionally found in the dataset of *H*. *ferrea* (S2 Fig).

Overall, the species diversity of phyllosphere fungi decreased with height; the smaller number of fungal OTUs showed greater dominance in the higher layers (Figs 6 and 7). This pattern was additionally found in the dataset of *H*. *ferrea* (Fig 6C and 6D; S2 Fig), wherein the number of samples was almost consistent every layer; therefore, the decrease in the species diversity of phyllosphere fungi was likely independent of that in the number of host plants.

### Vertical structure of phyllosphere fungal communities

**Fig 8:**
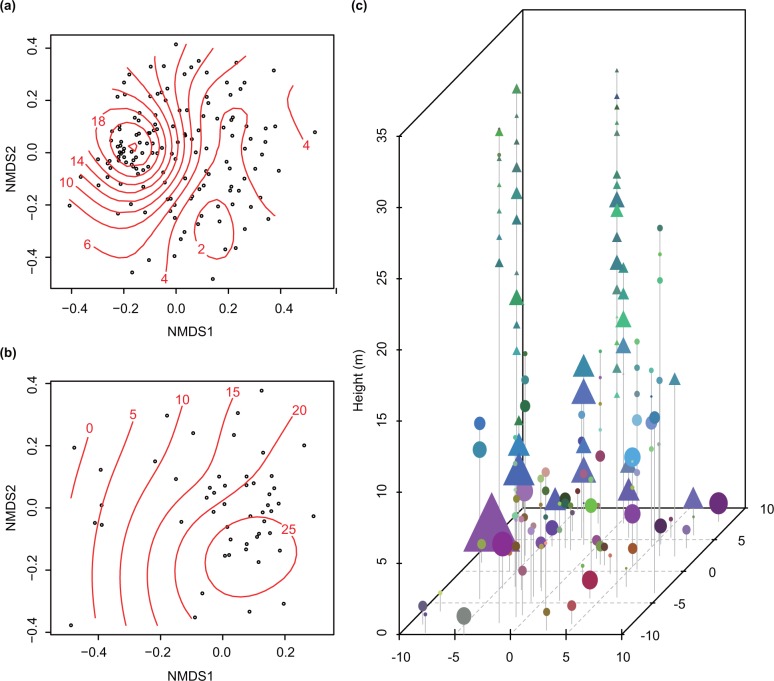
Spatial structure of phyllosphere fungal communities in a tropical evergreen forest. (a), (b) Two-dimensional NMDS plot of each fungal community based on Raup–Crick dissimilarity of fungal OTU compositions found in (a) all 144 samples and (b) the 51 *Hopea ferrea* samples. Red lines fitted on the ordinations indicate the height from the ground. (c) Three-dimensional mapping of 144 fungal communities on leaf samples. Each triangle and circle symbol indicates each fungal community of *H*. *ferrea* and other remaining plant species, respectively. Similarity of colors represents similarity of fungal OTU compositions. The size of the symbols represents the relative number of OTUs detected at each sampling points. The vertical lines connecting symbols indicate tree individuals.

**Fig 9:**
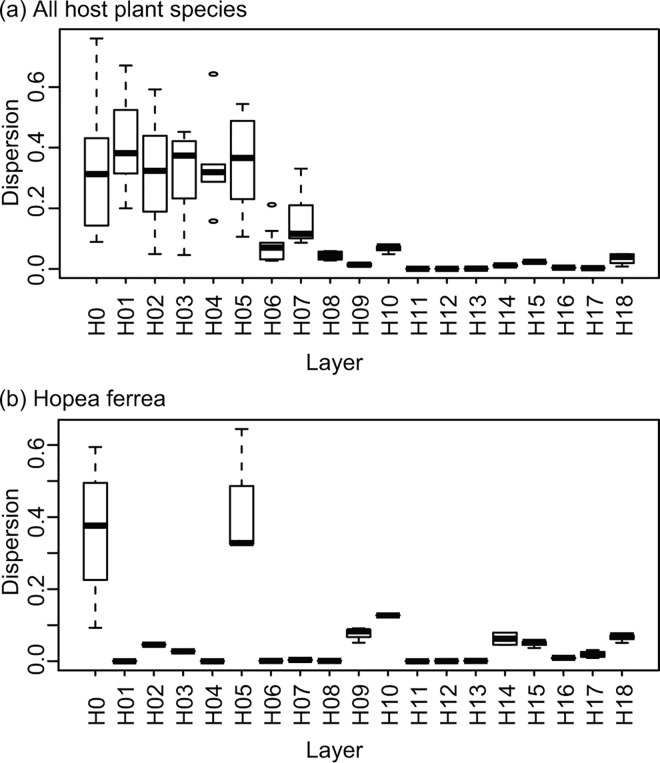
Dispersion of fungal community compositions among samples within vertical layers. Dispersion of fungal communities within a given layer is represented by the average distance between each fungal community and the centroid within a layer on the nonmetric multidimensional scaling plotting. Dispersions were calculated in the dataset of (a) all 144 samples and (b) the 51 *Hopea ferrea* samples.

In both datasets, NMDS ordinations based on the Raup–Crick dissimilarity and Jaccard similarity of phyllosphere fungal communities showed a clear change in OTU compositions along with the height (Fig 8A and 8B; S3 Fig). The second axis (NMDS2) in the dataset of all plant species and the first axis (NMDS1) in the dataset of *H*. *ferrea* accounted for the dissimilarity of fungal OTU compositions along the vertical layers (Fig 8A and 8B; S3 Fig); therefore, the height was a relatively large explanatory factor for the spatial variability of fungal OTU compositions, particularly in *H*. *ferrea*. As shown in Fig 8C, in the upper layers (H12–H18), most fungal communities within a layer were depicted in similar green colors, indicating lower variations among fungal OTU compositions. Conversely, in the lower layers (H0–H05), the fungal communities, even within one and the same layer, were shown in different colors, indicating the higher heterogeneity of fungal community compositions across sampling points. This pattern was also supported by the observation that the degree of differences in fungal OTU compositions within a layer was larger in the lower layers (H0–H05) and smaller in the higher layers (H06–H18), although the pattern was unclear in the dataset of *H*. *ferrea* (Fig 9).

**Fig 10:**
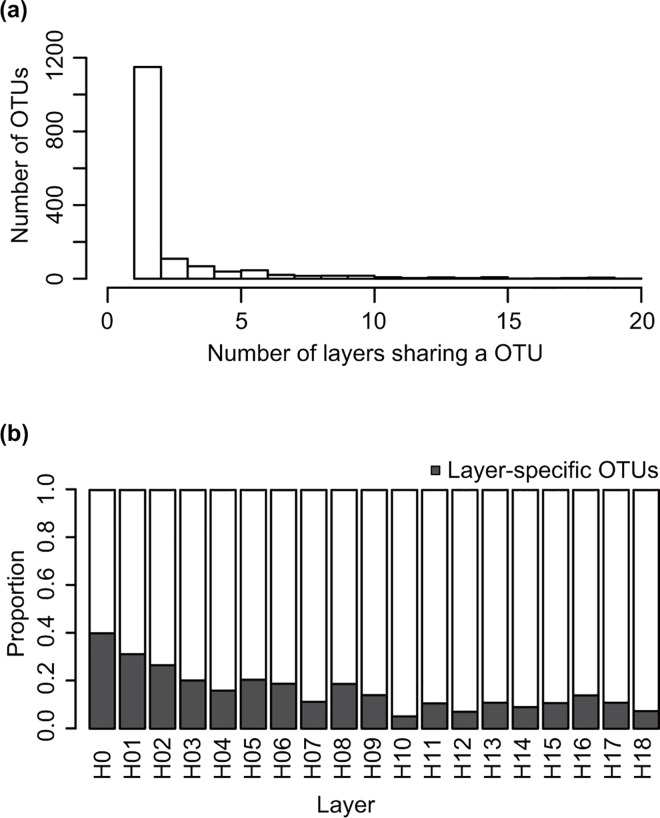
Distribution patterns of fungal operational taxonomic units (OTUs) across the 19 vertical layers in the forest. (a) The number of layers inhabited by 1,524 fungal OTUs. (b) Proportion of layer-specific OTUs among all fungal OTUs in each layer. The dataset of 144 samples collected from all 27 plant species was analyzed.

Each fungal OTU was found in 2.4 layers on average. Out of 1,524 OTUs, 1,150 OTUs (75.4%) were limitedly to one layer (Fig 10A) and defined as “layer-specific OTUs.” The proportion of layer-specific OTUs among fungal OTUs comprising the communities decreased with height; layer-specific OTUs occupied 39.9% on the layer closest to the forest floor (H0) but only 7.3–13.9% in the higher layers (H12–H18) (Fig 10B).

## Discussion

### Overall species diversity and taxonomic compositions of phyllosphere fungi

In the tropical seasonal evergreen forest, taxonomically diverse phyllosphere fungi are associated with various plant species from the understory to the canopy. Using next-generation sequencing, 1,524 fungal OTUs (890,710 reads) were obtained from 144 samples collected from 73 plant individuals of 27 species growing in a 314 m^2^ area × 34.4 m height. The number of fungal OTUs that were identified at species level was as small as 111 (7.3%) (Fig 3A). This could indicate that the majority of fungal species in the forest remain to be described by science or that the species have been described, but no ITS sequences are available for comparison. Other explanations could be that the sequence length (mean: 231 bp) may not be enough to infer taxonomic affiliations [54], or that a rDNA ITS1 region deposited in the databases for an individual species may not reflect the actual intraspecific genetic variation of this marker within a given species and inadequate to identify genetic variations among species [55]. The low proportions of identified OTUs also could be attributed to the conservative taxon assignment of the QCauto method, which provides a smaller number of identified OTUs but has been shown to assign more accurate taxonomic affiliations to OTUs compared to other methods [43]. Additionally to the many unidentified taxa, OTU richness estimated with Chao and jackknife species richness estimators exceeded the observed OTU richness in every layer (Fig 6A and 6B), indicating that additional fungal taxa remain to be sampled.

Ascomycota comprised a large proportion of the phyllosphere fungal communities in the forest (Fig 3B), which was consistent with the findings of previous studies targeting the fungi associated with plant leaves [1, 9, 10]. Within Ascomycota, Capnodiales and Pleosporales, which includes a large amount of taxa including parasites, endophytes, and epiphytes [56, 57], were the most abundant order in every vertical layer (Fig 4), indicating that they form the major component of the phyllosphere fungal flora in the forest. Among the 48 families found in the 1,524 fungal OTUs, no fungal families likely adapted specifically to the canopy in the forest (Fig 5). The phyllosphere fungi living in the canopy in the forest were composed of common fungi, many of which are known to be pathogens in plant leaves or woods [58, 59], saprobes and endophytes [60]. They have small conidia that enable them to spread over long distances [61] and thus are found in a wide range of ecological habitats in the temperate and tropical regions.

### Vertical gradient of species diversity and community composition of phyllosphere fungi

Within the forest, a clear shift in fungal species diversity and OTU compositions with tree height was found. In the layers close to the understory, species richness of phyllosphere fungi was high (Fig 6) and the community compositions were largely different among plant individuals (Figs 8C and 9). Along with increasing height, the observed and estimated species richness of phyllosphere fungi, especially the number of infrequent fungi, declined and the evenness in the frequency of occurrence increased (Figs 6 and 7). The OTU compositions of phyllosphere fungal communities also shifted (Fig 8A and 8B). Although the highest fungal diversity might be derived from the different ages or individuals of the 30 host trees in the H0 compared to the 43 trees in the layers above, the effect of height on the fungal OTU composition was also evident in the dataset without the H0 layer (data not shown) or that of the single host plant species, *H*. *ferrea* (Fig 8B). In the canopy, because of a relatively limited number of fungal taxa (Fig 6), similar fungal communities were observed among different plant individuals in a same layer (Fig 8C).

The current study showed that the species and compositional diversity of phyllosphere fungi in the canopy is likely low; the total abundance of fungal OTUs, particularly layer-specific OTUs in the canopy was lower than that in the lower layers. One reason for the limited diversity and spatial variability of fungal communities in the canopy may be the small number of individuals and species of host plants occupying the forest canopy; in H12–H18, the canopy vegetation was composed of 3.6 plant individuals on average, which belonged to the two species *H*. *ferrea* and *Melodinus cambodiensis* (Fig 2A; S1 Fig). Therefore, the fungi that specifically interacted with or preferred any other host plant species besides *H*. *ferrea* and *M*. *cambodiensis* was excluded from inhabiting the canopy. However, because plant flora in the canopy may be different according to forest structures [62], different spatial structures of phyllosphere fungal communities may be found in other forests.

Environmental conditions such as air temperature, humidity, desiccation, and ultraviolet radiation vary spatially and temporally within a forest [20, 21] and these micro-environmental conditions are known to influence fungal community compositions [63, 64]. The forest canopy is exposed to strong light, high temperatures, and strong winds [65]; therefore, the fungi inhabiting the canopy are likely to be tolerant to environmental extremes. The dispersal limitation of each fungal species additionally forms the fine-scale spatial structure of fungal communities [66]. Therefore, the vertical gradient of species diversity and compositions of phyllosphere fungal communities observed in this study might be affected by the change in the environmental conditions within a forest and/or dispersal limitation of fungi. Nonetheless, to determine the environmental factors underlying the distribution patterns in phyllosphere fungi, more physiological characteristics of each fungal species need to be studied.

In this study, the spatial structure of phyllosphere fungi (Fig 8C) and the habitat preference of fungi within a forest (Figs 4 and 5) were effectively revealed by the metabarcoding analysis. The frequency of occurrence and community compositions of phyllosphere fungi largely changed from the tropical forest understory to the canopy, even within a single plant species or individual. More than half (75.4%; Fig 10A) of the entire fungal OTUs were only detected from a single vertical layer (Fig 10A), while some fungal OTUs showed a very wide range of habitats (Fig 10A). Therefore, the entire species diversity of phyllosphere fungi should be evaluated along a wide spatial scale. Such an inclusive species richness of phyllosphere fungi across the different environmental conditions within a forest or a host plant species (Figs 6 and 7) can be informative to predict and conserve the species diversity of fungi in a global change scenario [9]. For example, we can have knowledge about how large changes in environmental conditions or host plants’ distributions affect the survival of a particular fungal taxon. Moreover, the variability of ecological functions in each fungus [1, 67] and specific plant–fungi interactions [5, 6, 68] should be addressed to understand factors underlying the distribution patterns of phyllosphere fungi.

## Supporting Information

S1 Data Command script for Claident v0.2.2015.11.19 used for filtering, clustering and taxonomy identification of OTUs. (SH)

S2 Data Consensus sequences of 1,524 fungal OTUs in the FASTA format. (FASTA)

S3 Data R command script and data files used for the community analysis. (ZIP)

S1 Fig The vertical change of plant vegetation from H0 to H18. The four major genera (*Hopea*, *Memecylon*, *Hydonocarpus*, and *Aglaia*) are indicated in color. Labels show the Tree IDs (see S1 Table).
(EPS)

S2 Fig Rank–abundance plot for fungal OTUs found in each vertical layer. For each OTU, the proportion of inhabiting samples out of all samples in the layer (frequency of occurrence) is shown against the rank of the number of inhabiting samples (frequency rank). A total of 51 samples collected from *Hopea ferrea* were analyzed (see also Fig 7).
(EPS)

S3 Fig Two-dimensional NMDS plot of each fungal community based on Jaccard similarity index of fungal OTU compositions. Two datasets were analyzed: (a) all 144 samples and (b) the 51 *Hopea ferrea* samples. Green lines fitted on the ordinations indicate the height from the ground (see also Fig 8A and 8B).
(EPS)

S1 Table Number of raw, filtered and retained reads and OTUs in each sample. (XLSX)

S2 Table Taxonomic affiliations identified using the GenBank and UNITE database. For the GenBank “nt” and the UNITE dynamic database, the QCauto method (Tanabe and Toju 2013) and BLAST implemented in QIIME (Caporaso et al. 2010) was used for identification.
(XLSX)

S3 Table The BLASTN results of 229 fungal OTUs whose taxonomic affiliations were not identified with Claident against the “Top 50 wanted” database in UNITE. (XLSX)
